# Urban tinkering

**DOI:** 10.1007/s11625-018-0611-0

**Published:** 2018-08-06

**Authors:** Thomas Elmqvist, José Siri, Erik Andersson, Pippin Anderson, Xuemei Bai, Pranab Kishore Das, Tatu Gatere, Andrew Gonzalez, Julie Goodness, Steven N. Handel, Ellika Hermansson Török, Jessica Kavonic, Jakub Kronenberg, Elisabet Lindgren, David Maddox, Raymond Maher, Cheikh Mbow, Timon McPhearson, Joe Mulligan, Guy Nordenson, Meggan Spires, Ulrika Stenkula, Kazuhiko Takeuchi, Coleen Vogel

**Affiliations:** 1 Stockholm Resilience Centre, Stockholm University, Stockholm, Sweden; 2 United Nations University International Institute for Global Health, Kuala Lumpur, Malaysia; 3 University of Cape Town, Cape Town, South Africa; 4 Australian National University, Canberra, Australia; 5 Das and Associates, Mumbai, India; 6 Kounkuey Design Initiative, Los Angeles, USA; 7 McGill University, Montreal, Canada; 8 Harvard University and Rutgers University, Cambridge, USA; 9 ICLEI, CBC, Cape Town, South Africa; 10 University of Lodz, Lodz, Poland; 11 The Nature of Cities, New York, USA; 12 University of Queensland, Brisbane, Australia; 13 START International, East Lansing, USA; 14 The New School, New York, USA; 15 KTH and Kounkuey Design Initiative, Los Angeles, USA; 16 Princeton University, Princeton, USA; 17 White Arkitekter, Göteborg, Sweden; 18 The University of Tokyo, IGES, Tokyo, Japan; 19 University of the Witwatersrand, Johannesburg, South Africa

**Keywords:** Urban infrastructure, Latent multi-functionality, Social–ecological–technological system

## Abstract

Cities are currently experiencing serious, multifaceted impacts from global environmental change, especially climate change, and the degree to which they will need to cope with and adapt to such challenges will continue to increase. A complex systems approach inspired by evolutionary theory can inform strategies for policies and interventions to deal with growing urban vulnerabilities. Such an approach would guide the design of new (and redesign of existing) urban structures, while promoting innovative integration of grey, green and blue infrastructure in service of environmental and health objectives. Moreover, it would contribute to more flexible, effective policies for urban management and the use of urban space. Four decades ago, in a seminal paper in *Science*, the French evolutionary biologist and philosopher Francois Jacob noted that evolution differs significantly in its characteristic modes of action from processes that are designed and engineered de novo (Jacob in Science 196(4295):1161–1166, 1977). He labeled the evolutionary process “tinkering”, recognizing its foundation in the modification and molding of existing traits and forms, with occasional dramatic shifts in function in the context of changing conditions. This contrasts greatly with conventional engineering and design approaches that apply tailor-made materials and tools to achieve well-defined functions that are specified a priori. We here propose that urban tinkering is the application of evolutionary thinking to urban design, engineering, ecological restoration, management and governance. We define urban tinkering as:

A mode of operation, encompassing policy, planning and management processes, that seeks to transform the use of existing and design of new urban systems in ways that diversify their functions, anticipate new uses and enhance adaptability, to better meet the social, economic and ecological needs of cities under conditions of deep uncertainty about the future.

This approach has the potential to substantially complement and augment conventional urban development, replacing predictability, linearity and monofunctional design with anticipation of uncertainty and non-linearity and design for multiple, potentially shifting functions. Urban tinkering can function by promoting a diversity of small-scale urban experiments that, in aggregate, lead to large-scale often playful innovative solutions to the problems of sustainable development. Moreover, the tinkering approach is naturally suited to exploring multi-functional uses and approaches (e.g., bricolage) for new and existing urban structures and policies through collaborative engagement and analysis. It is thus well worth exploring as a means of delivering co-benefits for environment and human health and wellbeing. Indeed, urban tinkering has close ties to systems approaches, which often are recognized as critical to sustainable development. We believe this concept can help forge much-closer, much-needed ties among engineers, architects, evolutionary ecologists, health specialists, and numerous other urban stakeholders in developing innovative, widely beneficial solutions for society and contribute to successful implementation of SDG11 and the New Urban Agenda.

## Introduction

The complexity and scale of global urban development over the next quarter of a century will demand radically new approaches in development towards global sustainability (Elmqvist et al. 2018). The world’s urban population has grown from about 200 million in 1900 to 3.9 billion in 2014 and will likely reach 6.4 billion people in 2050. Over the decades to come, rapid urbanization will therefore continue, particularly in Asia and Africa. In the mid-century, 65% of populations in developing countries and nearly 90% in the developed world will live in urban areas (United Nations 2014). Cities all over the world are even now experiencing multiple impacts from global environmental change, especially climate change and land degradation, and the degree to which they must cope with and adapt to these challenges will continue to increase. While traditional, narrowly focused, planned/engineered design strategies are clearly needed to avert or mitigate such impacts in certain well-defined contexts, they are unlikely to be able to meet the full social, environmental and economic goals of cities, most notably the need for healthy, sustainable urban environments (e.g. Sustainable Development Goal 11). Strong path dependency often dominates urban development, and investments in urban infrastructure designed to fulfill one function may frequently create lock-in situations that persist over decades or even centuries. We argue that a multidisciplinary, complex systems approach, inspired by evolutionary theory, can inform the strategic design of policies and interventions to deal with challenges of growing urban regions and uncertainties in various scenarios in reducing undesirable path dependencies. Such an approach would guide the design of new (and redesign of existing) urban structures, while promoting innovative integration of grey, green and blue infrastructure in service of environmental and health objectives. Moreover, it would contribute to more flexible, effective policies for urban management and the use of urban space.

In a landmark 1977 article, Nobel laureate François Jacob made note of the highly flexible, opportunistic character of evolutionary progress, which he labeled “tinkering” (Jacob 1977). Evolutionary tinkering involves the modification and molding of existing traits and forms, which occasionally results in dramatic shifts in function in the context of changing conditions. This contrast greatly with conventional engineering and design approaches that apply tailor-made materials and tools to achieve well-defined functions that are specified a priori.

Here, we explore the idea of Urban Tinkering as the application of this evolutionary approach to urban design, engineering, management and governance. We define urban tinkering as:

“a mode of operation, encompassing policy, planning and management processes, that seeks to transform the use of existing and design of new urban systems in ways that diversify their functions, anticipate new uses and enhance adaptability, to better meet the social, economic and ecological needs of cities under conditions of deep uncertainty about the future.”

We see the discourse around evolutionary tinkering as a source of inspiration on how to navigate an urban future dominated by deep uncertainty, complexity and non-linearity. The tempo and intensity of climate changes are not known, and a flexible approach to urban design must be entertained. In this sense, admitting to our ignorance of future conditions may be the most intelligent design assumption. Urban tinkering is relevant not only to the design and planning of future infrastructure, but also to management and use of existing and planned urban spaces/structures. With new understanding of the values of ecological services in cities (Elmqvist et al. 2015), there is growing interest in increasing the links among ecological structure and other layers of urban design. To achieve the latter, approaches that encourage repurposing, experimentation, and innovative usage of existing elements are key. If well designed, an urban tinkering approach may help to reduce costly lock-in situations by incorporating infrastructure with an inherent potential to change function where needed or desired (Table 1).

**Table 1:** Proposed differences between conventional approaches and tinkering approaches

	Conventional approaches	Tinkering approaches
Mode	Tailor-made materials and tools—one function, generic solution	Modified materials and tools, multiple functions, experimentation, playfulness, strongly anchored in local context, anticipation
Characteristics	Monolithic gray, costly to repurpose	Hybrid (blue–green, gray) potential to repurpose
Management	Often single subcomponent	Adaptive, multiple components
Capital	Mostly financial and manufactured	More human and social
Path dependence	Strong	Weaker
Risk approach	Linear thinking, high predictability fail-safe	Non-linear, high uncertainty safe to fail
Governance	More top-down	Adaptive both top-down and bottom-up, more participatory

Linkages between evolutionary theory and the built environment are far from new. Indeed, new understandings of adaptation in evolution have at times been inspired by observation of the built environment, architecture and design—the opposite of the relationship considered here. For example, in their highly influential paper “The spandrels of San Marco and the Panglossian paradigm,” Gould and Lewontin (1979) discussed how views of adaptation in evolutionary theory could be informed by insights into architecture and design, elaborating, in particular, on the ornamentation of spandrels—the tapering triangular spaces formed by the intersection of two rounded arches at right angles. Spandrels are the necessary architectural by-products of mounting a dome on rounded arches. In many buildings, such as the Cathedral of San Marco in Venice, Italy, they are occupied by exquisite paintings and illustrations, as elegantly described by Lewontin and Gould. The analogy here is that the spandrels were not designed de novo as a space for paintings and illustrations, but were a by-product with no specific function, later used to fulfill other functions. Similarly, in evolution, Lewontin and Gould argued that many organismal traits for which we try to ascribe an adaptive explanation may in fact have no adaptive value, or maybe secondarily modified (but see critical discussion in Queller 1995).

Theoretical linkages between the built environment and evolutionary theory are thus long-standing, and not only restricted to the natural sciences. Such approaches have been adopted in social sciences and engineering; for example, in accounting for technological change and the dynamics inherent in any social process (e.g. Dosi and Nelson 1994), or in understanding the patterns and processes of urban environmental change (Bai and Imura 2000). Specific applications often highlight the need for flexible policies and governance systems which facilitate bottom-up innovation (Kronenberg and Winkler 2009). Naturally, evolutionary approaches are also characteristic of analyses of social–ecological systems which explicitly assume co-evolution and mutual dependence of social and ecological components. The shift in thinking (with respect to dominant paradigms) needed to implement such approaches implies the need for a concomitant shift in the values of key actors and those of society at large.

We emphasize that the use of evolutionary insights in this paper is but a lens. We acknowledge the many obvious differences between urban development and evolving biological systems, such as the effects of human foresight and anticipation, innovation, and dissemination of ideas over large spatial scales. Such features may help to reduce the high transaction costs often observed in evolution in biological systems (i.e., high rates of extinction). This is not to suggest that tinkering emphasizes the economic efficiency central to dominant neo-liberal economic paradigms. Quite the opposite: tinkering allows for redundancy, diversity and complexity, and emphasizes precautionary repair and replacement, all of which favor the efficient functioning of a system as a whole, but not necessarily its individual processes. Indeed, the efficiency of a given social or economic process must be considered in the context of other processes necessary to its execution, and more generally, with respect to the functioning of the whole system.

The definition of urban tinkering adopted here relates closely to concepts already familiar in urban development, e.g., urban sustainability experiments and transitions, urban system innovations, adaptive management, ecosystem-based adaptation to climate change, nature-based solutions, and urban experimental labs (see e.g. Elmqvist et al. 2018; Bai et al. 2010). Urban tinkering may also be viewed as a conceptual cousin to “urban acupuncture” (Lerner 2014), “tactical urbanism” (Garcia and Lydon 2015) and similar ideas.

In our interpretation, however, tinkering includes some dimensions not captured by these other concepts; in particular, it explicitly stresses a social–ecological–technological complex systems perspective on the multi-functionality of new and existing urban structures, developed through collaborative engagement and analysis with a range of actors. Although urban tinkering in some ways resembles a combination of adaptive management and adaptive governance, it adds an important proactive dimension, anticipation, to these more reactive approaches. In addition, tinkering implies a dimension of curiosity and playfulness in experimentation and repurposing urban systems often lacking in other approaches (Table 1 and examples in “Box 1”).

## Box 1: Examples of urban tinkering in the city and beyond

**Fig. 1:**
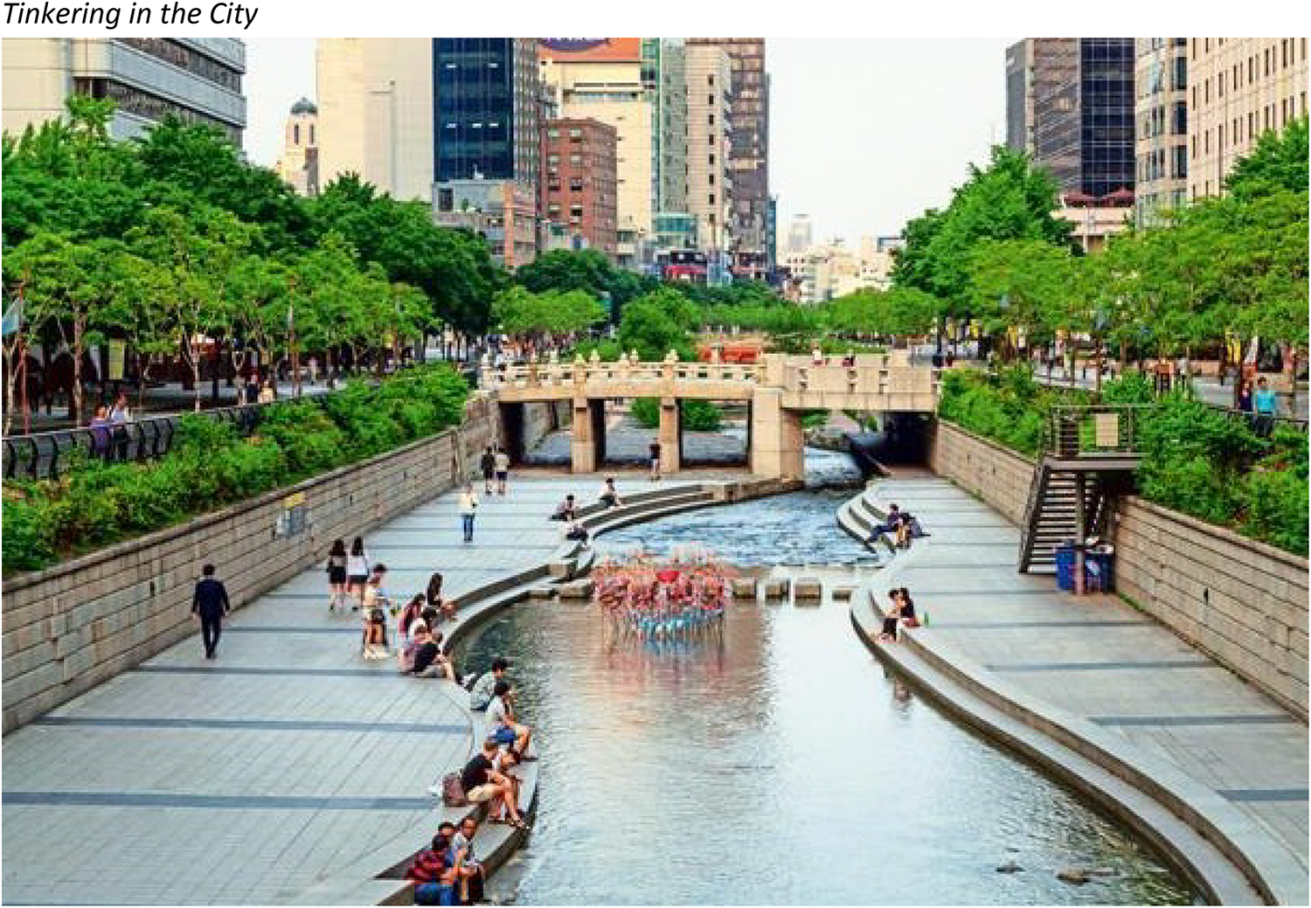
The Cheonggyecheon river after massive reconstruction and removing of a highway in Seoul

*Tinkering in the City (Fig.* *1**)*

The *Cheonggyecheon* (translated as “clear valley stream”) River runs through downtown Seoul. Originally free-flowing, it was covered over with concrete during a period of rapid urban development in the mid-20th century; an elevated highway was constructed overhead in the 1970s. This served as a main artery for transportation in Seoul, but the local neighborhood suffered increasing isolation, pollution and dilapidation, and by the 1990s, it was clear that structural issues would require major repairs to the road infrastructure. Led by then-Mayor Myung-Bak Lee, the city went in a different direction, consciously prioritizing human–nature relationships, public space, and cultural and historical values over further development of grey infrastructure. From 2003 to 2005, the roads covering the river were demolished, the river itself was restored, and a natural public space was created on its bounds. At the time, the decision was controversial, as many anticipated major traffic problems and economic losses for surrounding businesses. Indeed, among crucial aspects of the redevelopment were public–private partnerships to coordinate activities, simultaneous development of public transit infrastructure to mitigate decreased street flow, and support for local businesses during the period of reconstruction. The Cheonggyecheon river redevelopment has had major benefits not only for local economies, but for the health of residents and visitors, the quality and biodiversity of the local environment, and the wellbeing of the millions of annual visitors who visit its banks. It has led to the reintroduction of dozens of species of plants and animals, a reduction in local heat of up to 5 °C, and a 35% reduction in particulate air pollutants, while increasing ridership on public transit and removal of automobiles from streets.^1^ The project is not without critics: some say the redevelopment failed to provide sufficient community consultation; it has led to gentrification and loss of livelihood for some groups of resident workers; and the artificial watercourse has had persistent problems with algae and consequent budget overruns^2^ Nevertheless, it seems clear that Cheonggyecheon has been an essential element in changing the attitudes of residents of Seoul towards greater environmental consciousness.

*Tinkering in the hinterland*

Urban tinkering is not limited to the urban space itself, as tinkering approaches in the surrounding environment can also have significant effects for urban dwellers. In Japan, areas known as Satoyama lie on the outskirts of cities and farming villages. These landscapes of secondary forest border the lowlands and the hilly uplands which serve as sources for fuel and locations for farmed forest. The late 1960s saw drastic changes for Satoyama, as they became targets for large-scale housing development plans known as “newtowns” adjoining larger cities like Tokyo, Osaka and Nagoya. Several factors promoted this shift: (1) Satoyama were often retained as secondary spaces for agriculture in peri-urban areas and easily converted to built-up areas; (2) modern civil engineering technology made it possible to level the rough and hilly areas; (3) infrastructure development, including roads and railways, brought such areas within commuting distance of cities.

As Satoyama rapidly transformed into residential areas, local communities began to advocate more strongly for their conservation. Indeed, Satoyama were highly valued as part of the local natural environment, despite the ongoing changes. More recently, a growing academic movement emphasizes the multiple ecosystem functions—such as preservation of biodiversity, mitigation of urban heat-island effects, recreation and others—fulfilled by these spaces. Urban–rural interactions with the farming villagers who have traditionally managed Satoyama add further impetus to the demand for conservation.

In the twenty-first century, the Japanese population is declining in number and ageing dramatically. The speed of urbanization is also slowing dramatically such that major cities and adjacent Satoyama are unlikely to see any further large-scale residential development. More than ever, Satoyama will need to fulfill multiple functions. One particularly valued function is that of enhancing the spiritual and cultural welfare of an ageing society, and with little likelihood that Satoyama will be converted to residential areas, they are a prime subject for tinkering approaches. For example, they may serve as a stable repository of natural capital in the creation of a framework for urban green infrastructure, potentially contributing to the creation of ecological networks in cities, and to urban blue infrastructure, as in the case of rivers flowing from Satoyama to urban areas (Takeuchi et al. 2016).

Urban tinkering is perhaps most useful and easily applied in rapidly urbanizing regions of developing countries, harnessing social and human capital for innovation in informal settlements. According to the United Nations, two out of three people will live in urban areas by 2050 (United Nations 2014), but most of this urban growth will take place in Asia and Africa, where growth rates will average 3–5% per year (United Nations 2014). Africa’s urban population is already larger than the total population of North America, and—as in other urbanizing areas around the world—African cities are at significant risk for urban sprawl. Among its many other impacts, urban sprawl strongly influences city planning, as planners face the challenge of effectively updating and developing land use plans. Within such contexts urban tinkering, which allows for greater levels of flexibility and adaptability in planning, has an important role to play. Informality is common in most African cities (Myers 2011), especially where rapid urbanization outpaces the ability of planners to keep up. An explicit urban tinkering approach could to some extent counter this lag, allowing for the function of urban structures and processes to shift naturally on the basis of local needs and opportunities. While this cannot obviate the need for resources and services, it may allow for more efficient harnessing of the natural initiative and innovation often observed in informal settlements.

## Urban tinkering, architecture and design

Urban tinkering has close parallels in the creative design disciplines, among them architecture (including landscape architecture) and design. Architects and designers use well-established methods for creating solutions integrated into their unique social/physical/ecological context (Glanville 2007). Designers do not regularly communicate with ecologists, but the different training and vocabulary of these disciplines can be merged for a wider perspective to address new urban landscape needs (Palazzo and Steiner 2011). This will require a professional interplay that is atypical and can be tense (Handel 2014). Urban tinkerers can learn from such approaches, applying their strengths in other contexts while avoiding their shortcomings.

In simultaneously addressing the potentially conflicting demands of diverse stakeholders, architects develop designs through iterative feedback cycles (e.g. Kennedy-Clark 2013; Zimmerman 2010). Each cycle of design and testing provides new information for future revision towards greater synthesis (Amiel and Reeves 2008). Feedback cycles often engage a variety of stakeholders to provide more diverse perspectives on proposed design outcomes. However, the feedback cycles so critical to integrated design typically end before the project exists in the real world. In contrast, tinkering extends this feedback process indefinitely throughout the life of the project, through a process of ongoing critical reflection and revision by stakeholders. Additionally, critique in design approaches is typically based on the imagined outcomes of a design process. This means it is limited by the imagination, foresight and empathy of the designer. Urban tinkering expands this critique from theory into practice based on the lived experience of those involved.

Such ‘live editing’ approaches should not be seen as a substitute for foresight. While testing urban infrastructure amid real-life complexity is far more rigorous than imaginative/hypothetical testing alone, it also requires large investments of time and resources. Where foresight is possible, it is far more efficient to test proposals ‘on paper’ before committing resources to build infrastructure—it is easier to move a line than a wall. Architects routinely develop a design through many hundreds of iterations using sketches and models. This is where informed human foresight and anticipation is an important addition to the evolutionary process. Many existing professional methods for envisioning and shaping future outcomes will remain important for urban tinkering, including critical reflection, scenario modeling and stakeholder critique.

In our vision, tinkering is not confined to the very local scale of, e.g., houses and neighborhoods, but could well include larger spatial scales as whole cities or regions (see “Box 1”). Some critical urban functions (e.g., mobility and water management) are best addressed at larger scales, and cumulative adjustments of smaller-scale components can serve as the basis for systemic transformation. A tinkering approach could, for example, shift transportation networks or storm water systems into more modular structures where sub-components have higher autonomy and thus lend themselves more easily to experimentation. So many urban areas are coastal; with rapid sea level rise effecting regional centers, solutions for only local landscapes will be overwhelmed by impacts on adjacent areas. Only a wide-scale solution can be effective. There is also a large untapped potential for combining subsystems (e.g. transport, information, or green infrastructure) of the larger social–ecological–technological system such that, under changing conditions, they carry out old roles in new ways or take on new ones.

At larger scales, of course, the potential complexity of tinkering approaches increases, and coordination becomes more necessary. Larger-scale tinkering with systems like interstate rail and integrated power grids requires support from large-scale actors like regional and state governments. To serve as a comprehensive approach for whole city-regions, urban tinkering must combine agile, user-driven, bottom-up approaches with far-sighted, expert-driven, top-down approaches (see Table 1).

## Principles of urban tinkering

**Fig. 2:**
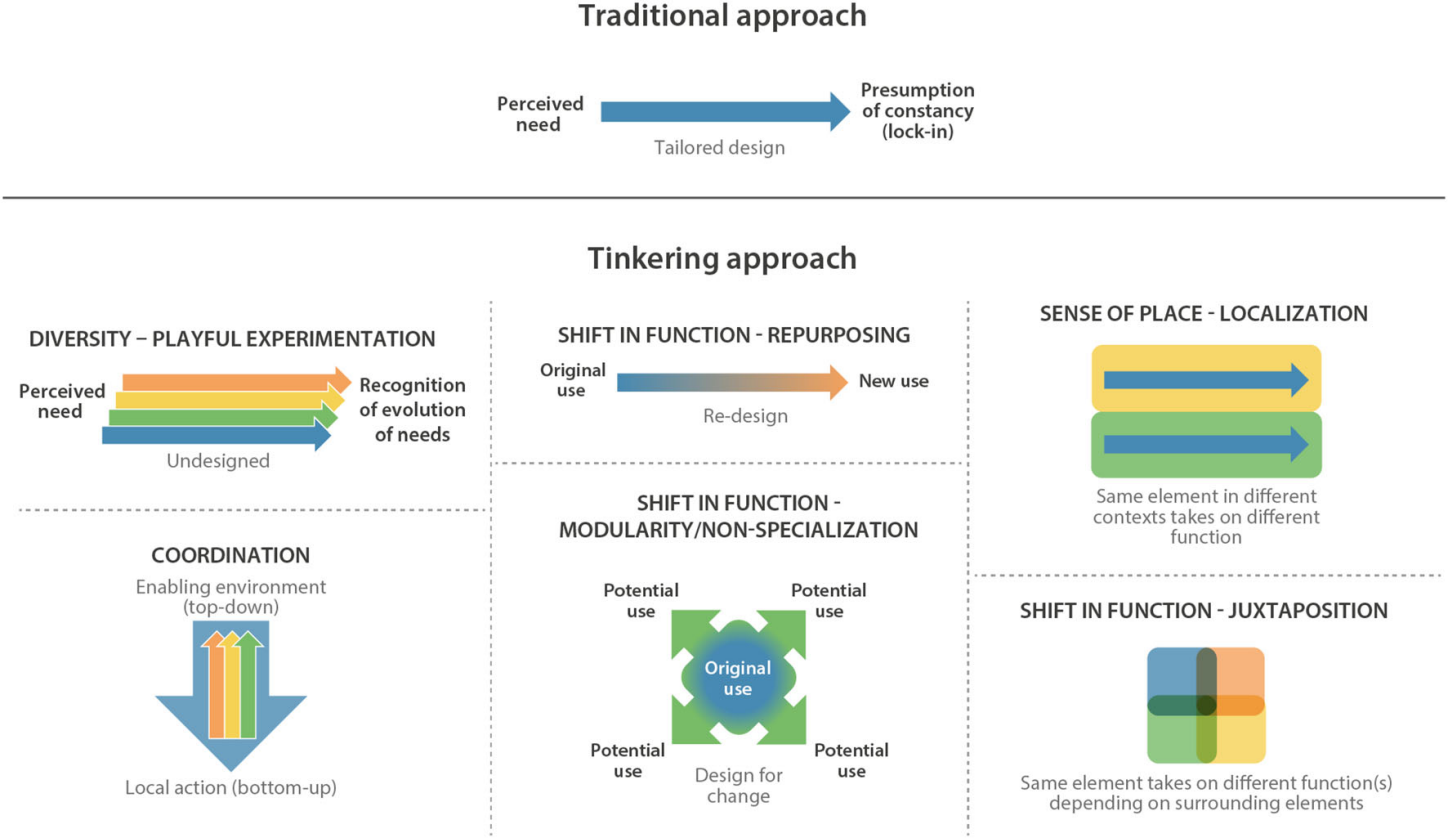
A linear traditional approach compared to the multifacets of tinkering approaches

Tinkering approaches can generate inspiration and new ways of thinking in a fragmented urban world characterized by deep uncertainty, complexity and non-linearity, contrasting with many current more linear views (Fig. 2).

The authors of this text span a diverse disciplinary background, including professional engineering, design, architecture, political science, evolutionary ecology, health and urban practice. Based on our collective experience, we propose six cross-cutting principles for successful tinkering.

## Diversity of approaches

To generate improved outcomes, tinkering should embrace experimentation; i.e., it must generate a diversity of approaches to existing challenges (Fig. 2). This can be accomplished via permissive policy or regulation for the use of space or existing elements of the built environment or through the a priori design of infrastructure adaptable to multiple functions (see example in “Box 2”). Most experiments will fail or achieve less-than-optimal outcomes (just as with evolution itself), yet the aggregate result of experimentation is the progressive discovery of improved function. The tinkering approach also constitutes a strong argument for equity, as the inclusive participation of diverse stakeholders—including those that are marginalized from mainstream debates—will contribute to the requisite diversity in approaches. To draw further parallels with the living world, it seems likely that cities, the sites of relentless small-scale experimentation, will mirror ecological systems in progressing along characteristic pathways of maturity in development—i.e., ecological succession. Increased attention to the feedback loops involved in this process, and a focus on understanding collective, emergent changes in the structures of urban components over time and the underlying systemic features that favor one over another may help inform the type, location or scale of desirable tinkering approaches.

## Box 2: The Corniche, Dakar, Senegal

In Dakar, natural spaces and recreational areas represent an aspiration for the growing middle class, yet have been steadily decreasing. The few existing green spaces have been poorly maintained, and are therefore relatively unappealing. To make the city more attractive to tourists and knowledge-based businesses and address citizens’ aspirations for open spaces for sport and recreation, the city began to explore options for recovering or creating natural spaces. One such area is the Corniche, the stretch of road and public space along the Atlantic coast; previously considered unsafe, it was redesigned in 2008 to become a more engaging multi-functional place, featuring monuments, arts, sport, walking tracks, hotels, shopping malls, museums, etc. The Corniche’s rebranding was essentially imposed by economic concerns of the city government, which aimed to extend adjacent hotel and health center infrastructures. Without a substantial reconceptualization and redesign, the Corniche would have remained unattractive, such that economic redevelopment efforts would have been unlikely to have achieved their potential. The vitalization of the Corniche depended on embracing a diversity of uses, including amenities such as running and cycling tracks, new sports equipment, public space managed by the municipality, palm tree plantations, spaces for children, art galleries, concert spaces, and shopping malls. Today, places such as Porte du Millenaire, Place du Souvenir, the Sea Plaza complex, and the Divinity Mosque are well known and well used by citizens and visitors.

## Shift in function

Often, tinkering approaches will lead to reimagined uses for existing urban elements (Fig. 2). To some extent, this is possible even with highly specialized elements (see example in “Box 3”). However, there will also be benefits to incorporating relatively unspecialized elements in urban design, to explicitly allow for multiple or shifting uses. For example, accessible public spaces are widely recognized as a critical feature of healthy cities, in part because they can be used by a wide variety of stakeholders to provide a diversity of social, economic, cultural and environmental services (see “Box 2”). There may be a role, too, for modular mobile structures that can be adapted to different uses or easily removed, replaced or combined according to need. Based on the principle that nothing is useless, old shipping containers, for example, have been used for everything from living spaces to restaurants to hotels to sanitation facilities to hospitals. For such outcomes to be successful, as with many of the tinkering approaches, participation among diverse stakeholders is key: one must draw from many points of view to give birth to novel ideas, escaping the constraints of tradition or common use. Playful imaginative experimentation, in a tinkering approach, can be useful in identifying valuable shifts in function.

A universal opportunity for urban tinkering can be on sanitary landfills which are infrastructure features worldwide. These sites are defined as engineering solutions to solid waste but have the potential to add value to many urban needs. The vast landforms can be sites of ecological structure, social amenity spaces (sports, family gatherings, urban agriculture) if the planning perspective can be changed. Each landfill site has a different potential, constraints by soil quality, adjacent land-uses, and economic needs, but “engineering” as the typology may be discounting the land’s highest value. Experimentation with different, new end-uses can bring new values to land parcels often considered derelict (Handel 2013).

## Box 3: The high line, New York City, USA^3^

Originally a train line, the High Line was operational from 1934 to 1980, until made obsolete by increasing interstate truck shipping. Community activism kept it from demolition over the succeeding two decades, until, in the late 1990s, a new group (“Friends of the High Line”) succeeded in marshaling support for its renovation and reuse as a public space. Opened in successive stages from 2009 to 2014, the High Line features art, commercial uses, and biodiverse green space managed using sustainable practices—including composting and integrated pest management (Friends of the Highline, http://www.thehighline.org/about/). The project attracts significant pedestrian traffic and tourism, offers multi-functional space for cultural and social events and has contributed significantly both to local revenue and to urban renewal in surrounding neighborhoods. As with other such efforts, it is not without controversy: local gentrification and a more homogeneous ethnic profile of visitors than for other sites, among other issues, have led to criticisms. Still, most agree that the High Line has led to a significantly more vital local space, while contributing to health and more pro-environmental attitudes among New Yorkers (Reichl 2016).

## Sense of place

Solutions obtained through urban tinkering are highly local, reinforcing the importance of place-based methods and the participation of local stakeholders (Table 1, Fig. 2). Often, a new function will arise out of the novel juxtaposition of otherwise familiar elements—by definition a local phenomenon (see example in “Box 4”). Juxtaposition need not be merely physical, but can involve linkages which produce its equivalent in social space. Opening urban spaces to tinkering approaches depends on a deep understanding of the relationships of people to places. Thus, for example, work emerging around the use and enjoyment of urban nature hints at the need or desire for a ‘facilitated’ nature experience (Brill 2017; Baigrie 2014). There is evidence to suggest that, in an urban setting, small signifiers that demonstrate the validity of multiple functions are extremely useful both in rendering hard infrastructure more accessible (for example a ladder into a dam to allow for swimming) and in making ‘wilderness’ more accessible (for example a toilet at a picnic site, or a bench). Small interventions, ‘signifiers’, small acts of ‘tinkering’, can serve to make urban features more accessible and potentially more equitable (and just). Indeed, such acts can expand the sense of ownership and belonging and allow for the kind of civic partnerships that can be useful in managing cities, particularly those that face fiscal constraints.

Remnants of past ecological structure and function exist in many urban centers, often in interstitial areas surrounded by large commercial or residential zones. These can be celebrated as mementos of preexisting ecologically functioning landscapes, and serve as reminders, not just of nature lost, but of the potential to restore lost landscape functions for a healthier future. People respond to the experience of urban nature as a guidepost to a most useful landscape (Lerner 2018).

## Box 4: Kibera, Nairobi, Kenya (Fig. 3)

Kibera is Kenya’s largest informal settlement. Along with other non-formal settlements in the city, it provides unique opportunities for new urban development structures and innovations that harness the social, economic and political capital of informal residents. Such initiatives can be interpreted as applying a tinkering approach. Examples include the unexpected re-utilization of existing urban elements by residents—such as the repurposing of an above-ground government sewer line in Lindi, Kibera, as an elevated footpath connecting two major access points into the settlement—or the localization of urban infrastructure, as evident in the design and installation of an aerial water pipe system that improves access to clean water for thousands of residents across the settlement while reducing vandalism from local water cartels. Shifting infrastructure functions are a point of departure for design adaptability and innovation, allowing infrastructure elements to serve multiple (and often unrelated) functions that address context-specific challenges, making neighborhoods and cities more resilient to physical and environmental conditions.^4^ The lack of formal services and of space require an ingenuity of mind and body to create workable living environments. This often leads to incredible multi-layered and multi-functional uses of private and public space which enable diverse economic and social movements (Table 1). These ingenious solutions represent a precious pool of applied design-thinking which should inspire all types of designers, planners and engineers. A recognition of the value and limitations of local “tinkering” solutions can inform designed and non-designed processes at multiple scales.

**Fig. 3:**
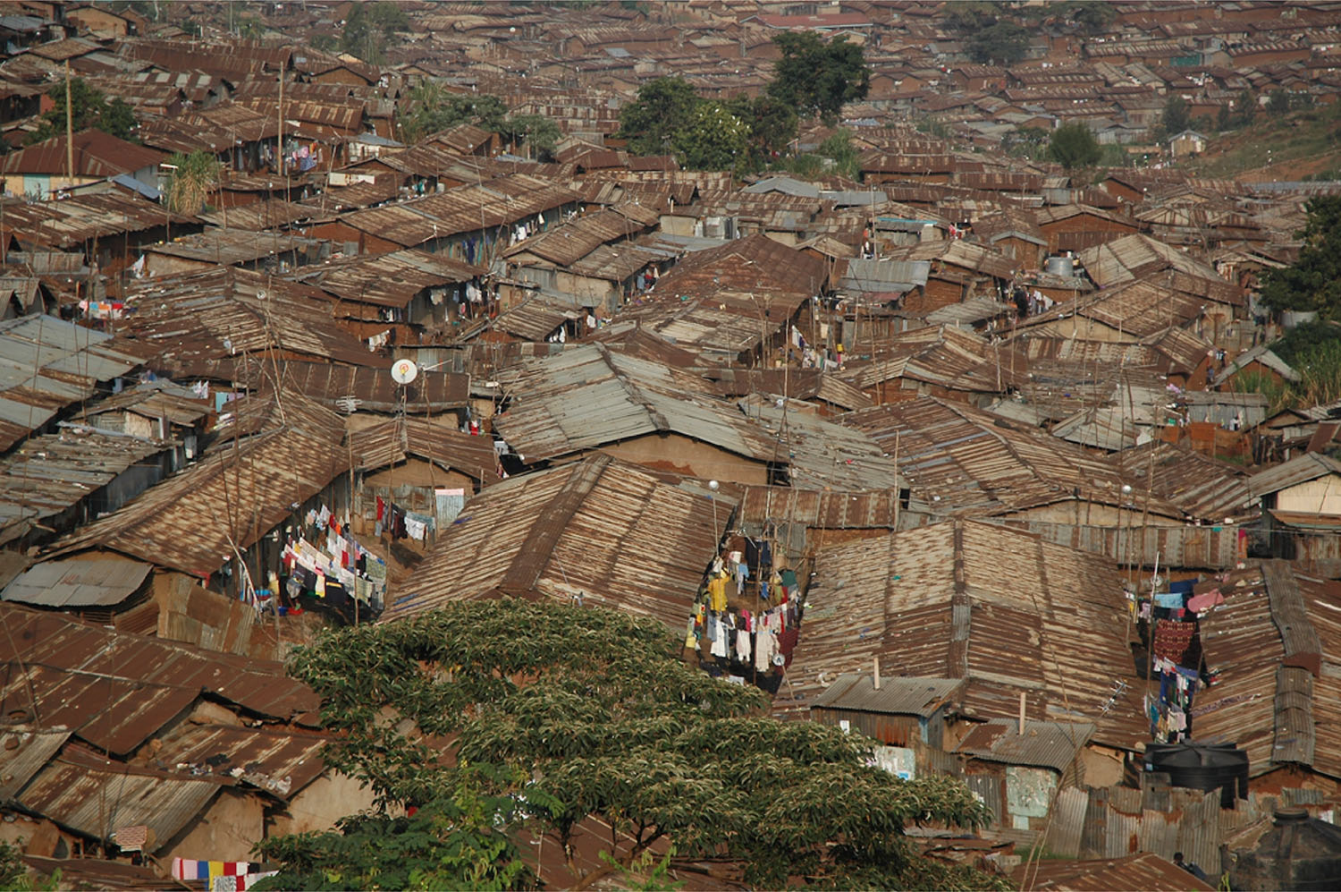
Kibera—high density but also with opportunities. Photograph: Joe Mulligan

## Coordination—adaptive management, adaptive governance—anticipation

There is room for both top-down (e.g., policy/regulation, large-project repurposing, structured experimentation) and bottom-up (e.g., local innovation, unstructured playful experimentation) approaches in urban tinkering, as well as for the combination of adaptive management, adaptive governance and anticipation. Critically, top-down approaches involving regulatory or policy action can fully complement bottom-up tinkering approaches, providing space, resources, opportunity and encouragement for local innovation (see example in “Box 5”).

## Box 5: The Cape flats nature program, Cape Town, South Africa (Fig. 4)

Launched in 2002, the Cape Flats Nature Program was jointly run by several conservation entities, with a view to building local capacity towards more inclusive conservation. It trained conservators, engaged local communities, and established joint and agreed management plans for local reserves. The program was radical in that while it sought to grow local conservators to lead and manage conservation spaces, this was always with a view to improving local social engagement in conservation practice and spaces. The program adopted a variety of reflective and reflexive practices, including listening to communities, hearing their stories of exclusion, their views and visions for green space in their communities, and involving them in planning and management strategies. Conservators were also encouraged to form their own communities of practice where they could share and reflect on failures and successes (Layne 2013). The Program embraced uncertainty in allowing a diversity of views to be heard; this in turn informed management processes and practices, often with unanticipated outcomes. A typical example was when neighborhood gangs approached the staff of one of the small reserves and asked to use their conservation education center for a meeting to broker peace. The types of engagements through the program meant the site was seen as communally owned, and yet unaligned—an unanticipated benefit of the open and inclusive processes followed. In this program an innovative and tinkering approach was adopted in revising historically conservative and exclusionary conservation practices to allow for a more fluid and reflective approach that allowed for unplanned outcomes

**Fig. 4:**
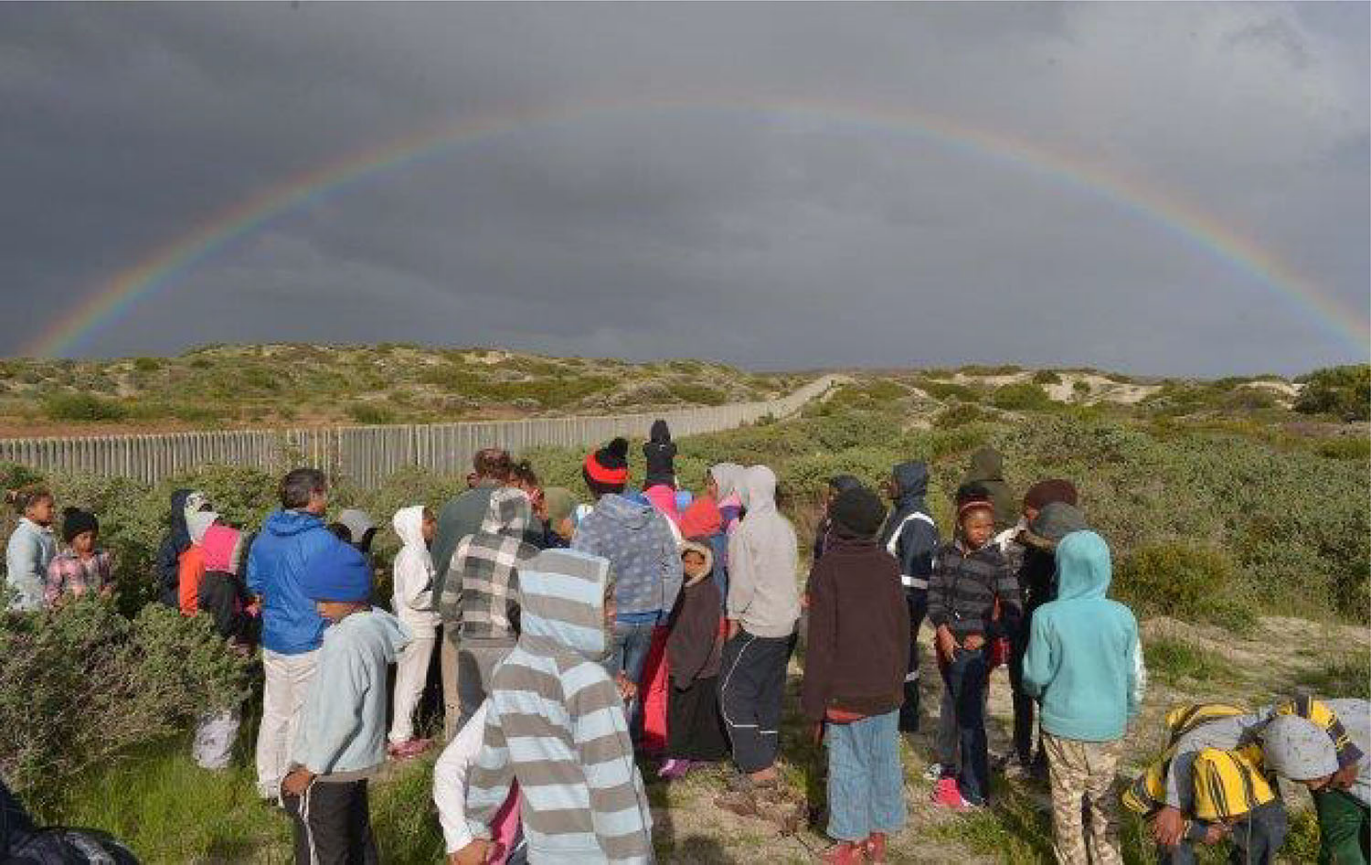
The Cape Flats Nature Project aimed to link stakeholders and conservation

*Evolution of community gardening*

Combination and recombination of different strands of knowledge, various land-uses, management practices and human needs are prominent features in the literature on adaptive co-management, in itself a practice where tinkering is quite common. For example, urban gardening provides an interesting case where traditions, practices and skills from one system have successfully been grafted onto another, and where there is potential for further tinkering (cf. Andersson and Barthel 2016). Different types of collectively managed urban gardens follow different parallel timelines and may—despite their differences—interconnect and influence the development of each other. Whereas the century-old allotment gardens of Europe tend to hold high levels of agro-biodiversity and well-established knowledge traditions (Barthel et al. 2010; Galluzzi et al. 2010), more recent community gardens seem more attractive to people with additional or alternative interests like political activity, back-to-work and rehabilitation programs, or small-scale business development. Nevertheless, these initiatives and the actors involved may over time become more nature oriented by exposing people who are not initially seeking engagement with nature to the added value of nature as a setting for different activities (Bendt et al. 2013; Holland 2004; Saldivar-tanaka and Krasny 2004). A faster way of getting there might be to seek linkages between the two systems of gardening, drawing on the rich diversity of social and ecological memory provided by allotment gardening. Similar to how allotment gardens once drew on knowledge and social memory borrowed from earlier kitchen gardening and agricultural communities (Barthel et al. 2010), community gardening could adopt practices and knowledge developed within a different context and somewhat different purpose and address anticipated challenges in the future.

## Extended time horizon

In biological systems subject to natural selection, the criterion for success is simple and obvious: survive and reproduce. In the context of a tinkering approach to urban development, success may be much less obvious, and will require new metrics.

For example, the success of a tinkering approach should be measured in the aggregate, rather than in individual projects, since new challenges and opportunities are identified throughout the life of a project. The necessary temporal scale may also vary significantly. On the one hand, a particular tinkering effort may be ephemeral but of great value, opening opportunities for further important downstream actions. On the other hand, quite a long interval may be needed to assess the value and efficacy of a tinkering paradigm.

This may be illustrated in the many urban areas near oceans, where the continuing sea level rise challenges infrastructure, residences, and coastal habitats. The large-scale urbanization near coastal zones creates a landform constraint where habitats (important for marine and well as upland ecological functioning) cannot migrate to higher ground when current sea–land edges are inundated. This is “coastal squeeze” where habitats are trapped and lost. However, without a reliable prediction of the degree and timing of sea level rise, tinkering with a variety of landform modifications may be necessary for ecological and economic sustainability (see “Box 6”).

Design of new landscape architecture projects must also recognize the rapid shift of vegetation zones that is now occurring (Grimm et al. 2013). Designs based only on current conditions denies the dynamic conditions facing today’s habitats. Stasis is not possible, and new approaches to tinkering with landscape design may be broadly necessary. Landscape elements, woodlands, meadows, shrublands, will shift in response to local climate and soil conditions if dispersal rates keep pace with climate shifts. A mosaic of habitats selected to reflect many possible future habitat placements can allow movement of species. In this sense stasis of a living landscape design is replaced by a suite of tinkering gestures, acknowledging that the habitats will reposition, in a currently unknown way. Again, admitting ignorance of the future advances the urgency of tinkering for resilient landscape structure.

## Box 6: Making space for the sea in urban areas during sea level rise

**Fig. 5:**
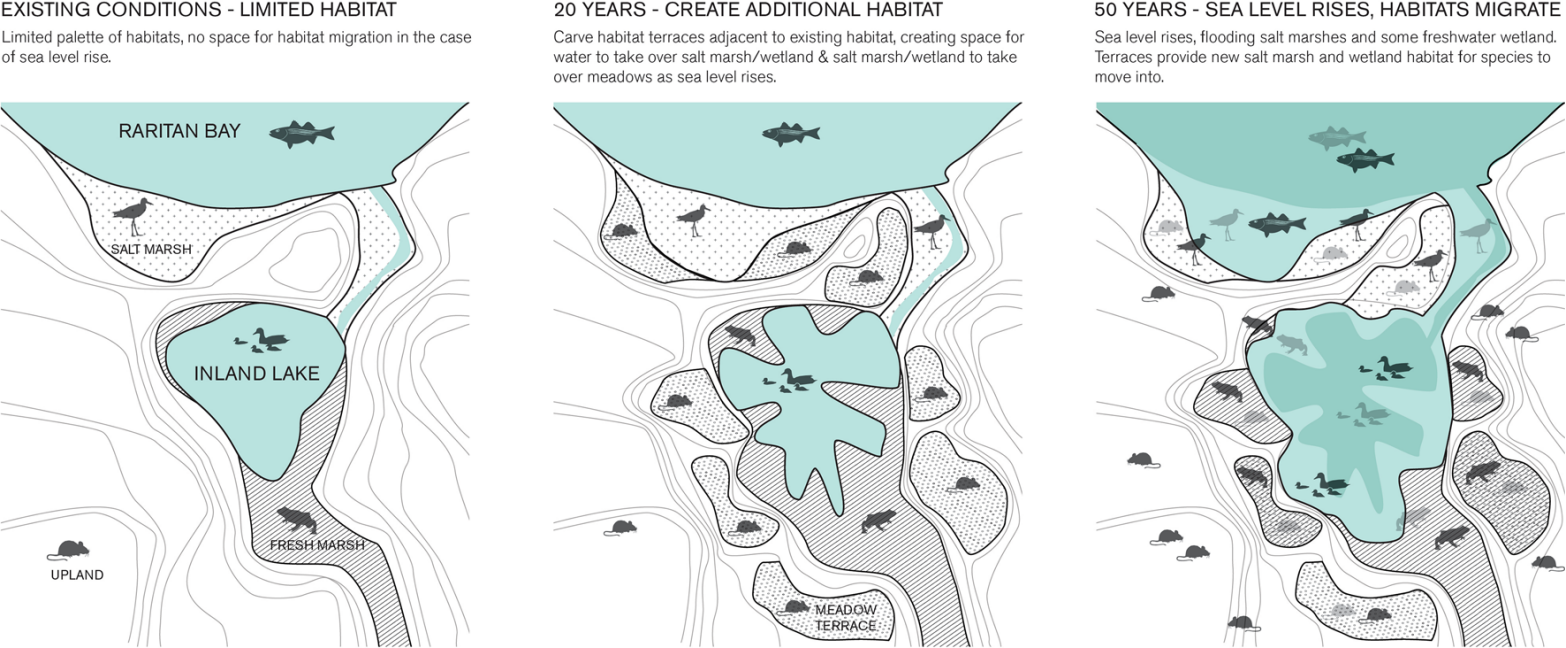
Modification of coastal landforms subject to future inundation by salt water maintains marine habitat area (Handel 2015)

Existing conditions (Fig. 5, left) have limited space for habitat migration as seas rise. Landforming tinkers with existing conditions (middle image) creating terraces available for marine/estuarine life as waters rise. If and when additional climate change occurs, the habitats revert to marine biodiversity, maintaining resources and services lost to the higher water level (Fig. 5, right).

There is no accurate prediction of the extent of sea level rise for many of our coastal cities. The only certainly is that the seas are rising. To maintain current land value and allow for protection of human settlements as well as ecological structure and function, local modification of the coastal landform may have wide application. In this example in the New York Metropolitan Area, a coastal town was heavily damaged by flooding during a recent hurricane, which more storms expected in coming decades. The sea level rise will flood existing intertidal habitats and economic drivers of the town’s tourist economy. Excavation of marginal lands into shallow basins will allow freshwater and edge habitats to be enlarged (Handel 2015). When sea levels rise, the quality of the water column changes slowly to estuarine, then marine. Marine biodiversity (invertebrates, fish, shorebird foraging habitat) moves inland by natural dispersal processes to replace lost areas. The position of the ecological and economic elements changes but are maintained. Experiments with the size, depth, and positioning of these urban manipulations can be tested, grounded in the movement biology of local species. Similar interventions, tinkering with the local landforms, can occur near barrier islands under threat, with ecological and social landscapes rearranged to new positions. The geography of environmental health changes but is not lost (Berger et al. 2016).

## Multi-systems approach

Conceptually, tinkering shares much with systems approaches, the two critical elements of which are analytic modes that can capture complex feedbacks, especially across sectors, and broad processes of engagement across stakeholder domains (e.g., public, private and civil sectors). Tinkering is a local manifestation of such approaches wherein actors from across society create joint experiments to achieve common goals. While the analytic component may be implicit in tinkering approaches, tinkering necessarily avails itself of feedback processes and draws upon cross-sectoral engagement.

A critical component to tinkering is social opportunity. Space (social, policy and physical) needs to be created for opportunities that allow for interventions or tinkering—opportunities to design in unconventional ways, to make innovative suggestions, to approach things differently. Significantly, these spaces, or gaps, can allow for champions (i.e., tinkers, in this reimagining) to emerge. People must feel empowered and unfettered to act, to try, to fail, to try again (“Box 7”). The literature suggests that champions emerge as a result of a particular set of personal characteristics (Howell 2005), but there need to be openings for these characters to emerge. The importance of individual citizens contributing to innovation, diversification and experimentation in, e.g., urban green space governance is often noted (Buijs et al. 2016; Mattijssen et al. 2017). Authorities need to allow flexibility with regard to mechanisms for bottom-up problem-solving, hence some system of flexible governance is required, as with urban commons (Colding and Barthel 2013) or so-called mosaic governance, which allows for context-sensitive planning, enhancing relationships between the diversity of landscapes and communities across cities (Buijs et al. 2016). The kind of social space that allows for this is often shunned as unconventional, time-wasting, or unproductive in the traditional economic sense. Inversely, the danger or blockage to useful redesign or rethinking around infrastructure and practice is restricted access. The sense of ‘license to act’—to engage, to fiddle with things—is critical to the process of tinkering.

## Box 7: Bandra beach in Mumbai (Das 2015)

**Fig. 6:**
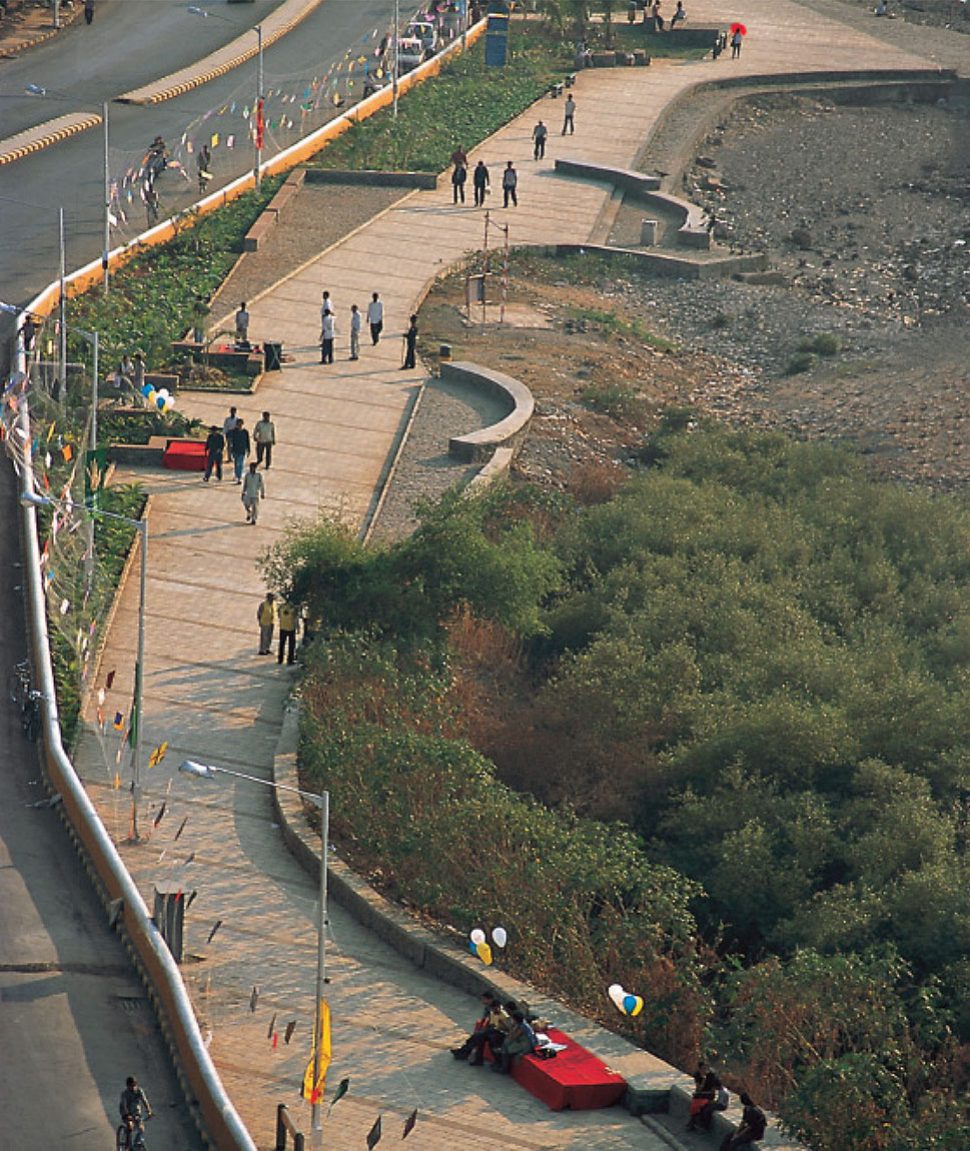
Bandra Beach development in Mumbai. Photo: P.K. Das

Mumbai has 16 km of beaches, which should provide an abundance of public open spaces to a city starved of it. But Mumbai’s beaches have been shrinking due to aggressive construction along the coast and consequent ecological damage, and restricted access due to highways, garbage, and private interests. The Bandra seafront development was a part of the larger conception of a movement for reclaiming Mumbai’s waterfronts, which in turn was a part of the idea of expanding Mumbai’s public spaces, which have dwindled to miserably low area as the city has grown (Fig. 6).

In many ways, the Bandra project’s success provided a focused example and direction for Mumbaikars across neighborhoods and the city of the need for citizen participation in influencing planning and development decisions relating to public spaces. Soon after its success Mumbai witnessed many similar movements. The new Mumbai Development Plan, which is likely to be released soon, has included a chapter devoted to open spaces, along with elaborate calculations and designations. This recognition can be attributed to public action and active engagement in documenting, protecting and preparing plans for their redevelopment. Without the targeted interventions of activists, the development of Mumbai would likely have continued on a trajectory of reduced open and green space, and compromised ecology and livability. The focused action in Bandra, led by a small group focused on the improvement of a specific place around a set of community values, served as an example of success and inspiration. If it could happen in Bandra—the reclamation of a space for community and improved quality of live—then it could happen elsewhere. The tinkering in Bandra served as a seed for a larger movement to give open space and community values a seat at the Mumbai planning table.

## Caveats and challenges to global sustainability

Finally, achieving the critical, but extremely challenging task of transforming social, economic, ecological, and technical infrastructure systems toward global sustainability in the long-term will require more than adding up combined tinkering efforts of cities. Although in our view, urban tinkering may have a tremendous potential to bring together fragmented dimensions of urban development, it is not a panacea. It is unlikely to, by itself, effectively address all the urban challenges we face, nor to deliver the kind of transformative change and at the scales and magnitude required to meet the sustainable development goals. It is also unlikely to completely displace conventional engineering from large-scale planned infrastructure.

Furthermore, no matter how transformative urban tinkering efforts are, we cannot assume that global sustainability and the successful implementation of SDG11 and the New Urban Agenda will be a granted as an end result. In fact, there are likely to be significant trade-offs, contestations, conflicts and unforeseen side effects and consequences of urban sustainability initiatives at all scales. To address these challenges, local and regional tinkering initiatives may need to be combined with a new globalization taking on a new face with a multipolar world developing, with thriving local and regional social, cultural and ecological diversity and governance, and where a new urban–rural regional integration is possible. Moving forwards requires flexibility, understanding of what determines learning, visions and imagination, and open-mindedness to deal with the unexpected, challenges and opportunities and deep uncertainties.

## Footnotes

1 1 Institute for Transportation and Development Policy case study. http://development.asia/case-study/revitalizing-city-reviving-stream.

2 See, e.g., The Guardian, https://www.theguardian.com/cities/2016/may/25/story-cities-reclaimed-stream-heart-seoul-cheonggyecheon.

3 Design Trust for Public Space (2002). Reclaiming the Highline. New York, NY: Ivy Hill Corporation.

4 https://www.theguardian.com/global-development-professionals-network/2016/oct/06/aerial-water-cartel-slum-kenya.
